# *N*-(2-Chloro-4-nitro­phen­yl)-2-nitro­benzamide

**DOI:** 10.1107/S1600536808006430

**Published:** 2008-03-12

**Authors:** Aamer Saeed, Shahid Hussain, Ulrich Flörke

**Affiliations:** a Department of Chemistry, Quaid-i-Azam University, Islamabad, Pakistan; b Department Chemie, Fakultät für Naturwissenschaften, Universität Paderborn, Warburgerstrasse 100, D-33098 Paderborn, Germany

## Abstract

In the title compound, C_13_H_8_ClN_3_O_5_, the dihedral angle between the two aromatic rings is 70.74 (6)°. The nitro groups of the Cl-substituted and benzamide benzene rings are twisted by 2.6 (1) and 31.3 (2)°, respectively. The crystal packing shows inter­molecular C—H⋯O hydrogen bonds that link mol­ecules into sheets stacked along [010].

## Related literature

For the biological activities of benzanilides and related compounds, see: Makino *et al.* (2003 ▶); Ho *et al.* (2002 ▶); Zhichkin *et al.* (2007 ▶); Jackson *et al.* (1994 ▶); Capdeville *et al.* (2002 ▶); Igawa *et al.* (1999 ▶). For related structures, see: Di Rienzo *et al.* (1980 ▶); Batsanov & Lyubchik (2003 ▶).
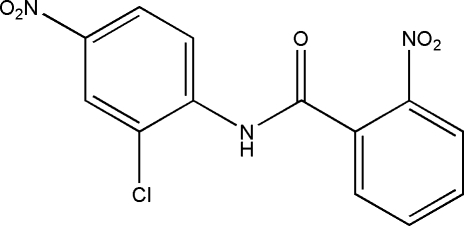


## Experimental

### 

#### Crystal data

C_13_H_8_ClN_3_O_5_
*M* *_r_* = 321.67
Orthorhombic, 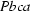

*a* = 7.8053 (9) Å
*b* = 13.8621 (17) Å
*c* = 24.101 (3) Å
*V* = 2607.7 (5) Å^3^
*Z* = 8
Mo *K*α radiation
μ = 0.32 mm^−1^
*T* = 120 (2) K
0.47 × 0.20 × 0.14 mm

#### Data collection

Bruker SMART APEX diffractometer
Absorption correction: multi-scan (*SADABS*; Sheldrick, 2004 ▶) *T*_min_ = 0.863, *T*_max_ = 0.956
21562 measured reflections
3111 independent reflections
2424 reflections with *I* > 2σ(*I*)
*R*_int_ = 0.053

#### Refinement

*R*[*F* ^2^ > 2σ(*F* ^2^)] = 0.043
*wR*(*F* ^2^) = 0.107
*S* = 1.04
3111 reflections
199 parameters
H-atom parameters constrained
Δρ_max_ = 0.39 e Å^−3^
Δρ_min_ = −0.23 e Å^−3^

### 

Data collection: *SMART* (Bruker, 2002 ▶); cell refinement: *SAINT* (Bruker, 2002 ▶); data reduction: *SAINT*; program(s) used to solve structure: *SHELXS97* (Sheldrick, 2008 ▶); program(s) used to refine structure: *SHELXL97* (Sheldrick, 2008 ▶); molecular graphics: *SHELXTL* (Sheldrick, 2008 ▶); software used to prepare material for publication: *SHELXTL*.

## Supplementary Material

Crystal structure: contains datablocks I, global. DOI: 10.1107/S1600536808006430/si2073sup1.cif

Structure factors: contains datablocks I. DOI: 10.1107/S1600536808006430/si2073Isup2.hkl

Additional supplementary materials:  crystallographic information; 3D view; checkCIF report

## Figures and Tables

**Table 1:** Hydrogen-bond geometry (Å, °)

*D*—H⋯*A*	*D*—H	H⋯*A*	*D*⋯*A*	*D*—H⋯*A*
C13—H13*A*⋯O1	0.95	2.24	2.848 (2)	121
C10—H10*A*⋯O4^i^	0.95	2.35	3.246 (2)	157
C11—H11*A*⋯O2^ii^	0.95	2.55	3.202 (2)	126

Symmetry codes: (i) 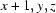; (ii) 
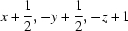
.

**Fig. 1.**
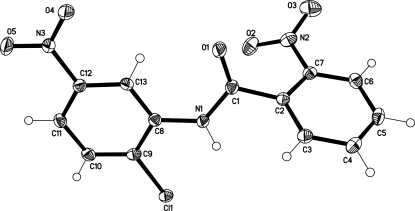
Molecular structure of the title compound. Displacement ellipsoids are drawn at the 50% probability level.

**Fig. 2.**
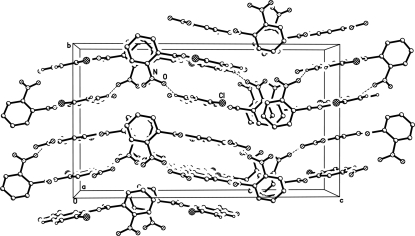
Crystal packing viewed along [100] with intermolecular hydrogen bonding pattern indicated as dashed lines. H-atoms not involved in hydrogen bonding are omitted.

## supplementary crystallographic information

### Comment

The benzanilide core is present in compounds with such a wide range of biological activities that it has been called a privileged structure. Benzanilides serve as intermediates towards benzothiadiazin-4-ones (Makino *et al.*, 2003), benzodiazepine-2,5-diones (Ho *et al.*, 2002), and 2,3-disubstituted 3*H*-quinazoline-4-ones (Zhichkin *et al.*, 2007). Benzanilides have established their efficacy as centroid elements of ligands that bind to a wide variety of receptor types. Thus benzanilides containing aminoalkyl groups originally designed as a peptidomimetic, have been incorporated in an Arg-Gly-Asp cyclic peptide yielding a high affinity GPIIb/IIIa ligand (Jackson *et al.*, 1994). Imatinib is an ATP-site binding kinase inhibitor and platelet-derived growth factor receptor kinases (Capdeville *et al.*, 2002).Benzamides have activities as acetyl-CoA carboxylase and farnesyl transferase inhibitors (Igawa *et al.*, 1999) The literature is full of the function of the 2-chloro-4-nitrophenyl group (CNP) and also structures of nitrobenzamide (NB) and related compounds (Di Rienzo *et al.*, 1980; Batsanov & Lyubchik, 2003). The aim of the present work was to combine CNP and NB in a single structure which is not well known in the literature.

Geometric parameters of the title compound, C_13_H_8_ClN_3_O_5_, are in the usual ranges. The dihedral angle between the two aromatic rings is 70.74 (6)°. The N2 nitro group is twisted by 31.3 (2)° from the plane of the C2–C7 phenyl ring, and the N3 group 2.6 (2)° from the C8–C13 plane, respectively. The crystal packing shows intermolecular C–H···O hydrogen bonds, from the Cl-phenyl group to both nitro groups. Details are depicted in Table 1. By these hydrogen bonds molecules are linked to endless sheets that are stacked along [010]. Additionally, stacking of molecules along [100] can be recognized. The intramolecular C13–H13A···O1 interaction is a common feature for this molecule with an almost planar O1–C1–N1–C8–C13 arrangement. The corresponding torsion angles are C8–N1–C1–O1 6.7 (3)° and C1–N1–C8–C13 - 7.6 (3)°, respectively.

### Experimental

2-Nitrobenzoyl chloride (5.4 mmol) in CHCl_3_ was treated with 2-chloro-4-nitroaniline (21.6 mmol) under a nitrogen atmosphere at reflux for 3 h. Upon cooling, the reaction mixture was diluted with CHCl_3_ and washed consecutively with aq 1 *M* HCl and saturated aq NaHCO_3_. The organic layer was dried over anhydrous sodium sulfate and concentrated under reduced pressure. Crystallization of the residue in CHCl_3_ afforded the title compound (84%) as white needles: IR (KBr) 3226, 1665, 1616, 1520, 1352 cm-1; 1H NMR (CDCl3, 400 MHz) ? 8.13 (d, J) 8 Hz, 1H), 7.81 (d, J) 8 Hz, 1H), 7.51 (dd, J) 8 Hz, 1H), 7.66 (dd, J) 8 Hz, 1H), 7.43 (d, J) 8 Hz, 2H), 7.36 (br s, 1H), 7.25 (d, J) 8 Hz, 1H); 13 C NMR (100 MHz) ? 164.7, 147.8, 134.6, 134.4, 132.7, 132.1, 130.3, 129.9, 129.3, 125.0. Anal. Calcd. For C_13_H_9_N_3_O_5_, C, 48.54; H, 2.51; Cl, 11.02; N, 13.06 found C, 48.12; H, 2.31; Cl, 11.3; N, 12.94.

### Refinement

Hydrogen atoms were located in difference syntheses, refined at idealized positions riding on the carbon or nitrogen atoms with isotropic displacement parameters *U*_iso_(H) = 1.2*U*_eq_(C or N).

### Crystal data

**Table:**

C_13_H_8_ClN_3_O_5_	*F*_000_ = 1312
*M**_r_* = 321.67	*D*_x_ = 1.639 Mg m^−^^3^
Orthorhombic, *P**b**c**a*	Mo *K*α radiation λ = 0.71073 Å
Hall symbol: -P 2ac 2ab	Cell parameters from 769 reflections
*a* = 7.8053 (9) Å	θ = 2.9–25.7º
*b* = 13.8621 (17) Å	µ = 0.32 mm^−^^1^
*c* = 24.101 (3) Å	*T* = 120 (2) K
*V* = 2607.7 (5) Å^3^	Prism, colourless
*Z* = 8	0.47 × 0.20 × 0.14 mm

### Data collection

**Table:**

Bruker SMART APEX diffractometer	3111 independent reflections
Radiation source: sealed tube	2424 reflections with *I* > 2σ(*I*)
Monochromator: graphite	*R*_int_ = 0.053
*T* = 120(2) K	θ_max_ = 27.9º
φ and ω scans	θ_min_ = 1.7º
Absorption correction: multi-scan(SADABS; Sheldrick, 2004)	*h* = −10→10
*T*_min_ = 0.863, *T*_max_ = 0.956	*k* = −18→16
21562 measured reflections	*l* = −31→31

### Refinement

**Table:**

Refinement on *F*^2^	Secondary atom site location: difference Fourier map
Least-squares matrix: full	Hydrogen site location: difference Fourier map
*R*[*F*^2^ > 2σ(*F*^2^)] = 0.043	H-atom parameters constrained
*wR*(*F*^2^) = 0.107	*w* = 1/[σ^2^(*F*_o_^2^) + (0.0553*P*)^2^ + 0.7975*P*] where *P* = (*F*_o_^2^ + 2*F*_c_^2^)/3
*S* = 1.04	(Δ/σ)_max_ < 0.001
3111 reflections	Δρ_max_ = 0.39 e Å^−^^3^
199 parameters	Δρ_min_ = −0.23 e Å^−^^3^
Primary atom site location: structure-invariant direct methods	Extinction correction: none

### Special details

**Table:**

Geometry. All e.s.d.'s (except the e.s.d. in the dihedral angle between two l.s. planes) are estimated using the full covariance matrix. The cell e.s.d.'s are taken into account individually in the estimation of e.s.d.'s in distances, angles and torsion angles; correlations between e.s.d.'s in cell parameters are only used when they are defined by crystal symmetry. An approximate (isotropic) treatment of cell e.s.d.'s is used for estimating e.s.d.'s involving l.s. planes.
Refinement. Refinement of *F*^2^ against ALL reflections. The weighted *R*-factor *wR* and goodness of fit *S* are based on *F*^2^, conventional *R*-factors *R* are based on *F*, with *F* set to zero for negative *F*^2^. The threshold expression of *F*^2^ > σ(*F*^2^) is used only for calculating *R*-factors(gt) *etc*. and is not relevant to the choice of reflections for refinement. *R*-factors based on *F*^2^ are statistically about twice as large as those based on *F*, and *R*- factors based on ALL data will be even larger.

### Fractional atomic coordinates and isotropic or equivalent isotropic displacement parameters (Å^2^)

**Table:**

	*x*	*y*	*z*	*U*_iso_*/*U*_eq_	
Cl1	1.13944 (6)	0.38512 (3)	0.446787 (18)	0.02271 (13)	
O1	0.49413 (17)	0.41462 (11)	0.39481 (5)	0.0317 (3)	
O2	0.6129 (2)	0.24007 (10)	0.33719 (6)	0.0374 (4)	
O3	0.4575 (2)	0.23191 (11)	0.26277 (7)	0.0428 (4)	
O4	0.38629 (18)	0.37084 (12)	0.57016 (6)	0.0388 (4)	
O5	0.54709 (19)	0.33143 (11)	0.63925 (5)	0.0345 (4)	
N1	0.7841 (2)	0.41233 (11)	0.40825 (6)	0.0221 (3)	
H1A	0.8816	0.4222	0.3908	0.027*	
N2	0.5594 (2)	0.27328 (11)	0.29309 (7)	0.0269 (4)	
N3	0.5267 (2)	0.35472 (11)	0.59071 (6)	0.0229 (3)	
C1	0.6395 (2)	0.41795 (13)	0.37682 (7)	0.0208 (4)	
C2	0.6749 (2)	0.43395 (13)	0.31581 (7)	0.0191 (4)	
C3	0.7458 (2)	0.52025 (13)	0.29780 (7)	0.0226 (4)	
H3A	0.7797	0.5675	0.3242	0.027*	
C4	0.7676 (3)	0.53806 (14)	0.24147 (7)	0.0253 (4)	
H4A	0.8179	0.5969	0.2296	0.030*	
C5	0.7163 (3)	0.47032 (14)	0.20255 (7)	0.0254 (4)	
H5A	0.7310	0.4831	0.1641	0.030*	
C6	0.6437 (2)	0.38416 (14)	0.21950 (7)	0.0237 (4)	
H6A	0.6073	0.3376	0.1931	0.028*	
C7	0.6253 (2)	0.36732 (13)	0.27579 (7)	0.0200 (4)	
C8	0.7961 (2)	0.39263 (12)	0.46521 (7)	0.0190 (4)	
C9	0.9581 (2)	0.37737 (12)	0.48863 (7)	0.0196 (4)	
C10	0.9792 (2)	0.35510 (13)	0.54414 (7)	0.0216 (4)	
H10A	1.0907	0.3443	0.5587	0.026*	
C11	0.8378 (2)	0.34852 (13)	0.57846 (7)	0.0211 (4)	
H11A	0.8497	0.3338	0.6168	0.025*	
C12	0.6785 (2)	0.36410 (12)	0.55510 (7)	0.0192 (4)	
C13	0.6534 (2)	0.38633 (12)	0.49987 (7)	0.0194 (4)	
H13A	0.5414	0.3971	0.4858	0.023*	

### Atomic displacement parameters (Å^2^)

**Table:**

	*U*^11^	*U*^22^	*U*^33^	*U*^12^	*U*^13^	*U*^23^
Cl1	0.0156 (2)	0.0279 (2)	0.0246 (2)	0.00115 (17)	0.00315 (15)	0.00015 (17)
O1	0.0189 (7)	0.0559 (10)	0.0203 (6)	0.0026 (7)	0.0019 (5)	0.0048 (6)
O2	0.0461 (10)	0.0290 (8)	0.0371 (8)	−0.0004 (7)	0.0064 (7)	0.0124 (6)
O3	0.0422 (10)	0.0316 (8)	0.0545 (10)	−0.0107 (7)	−0.0013 (8)	−0.0119 (7)
O4	0.0163 (7)	0.0739 (12)	0.0262 (7)	−0.0008 (7)	−0.0005 (6)	0.0062 (7)
O5	0.0305 (8)	0.0551 (10)	0.0178 (6)	0.0020 (7)	0.0025 (5)	0.0071 (6)
N1	0.0153 (8)	0.0339 (9)	0.0172 (7)	0.0003 (7)	0.0018 (6)	0.0034 (6)
N2	0.0255 (9)	0.0226 (8)	0.0325 (9)	0.0008 (7)	0.0081 (7)	−0.0029 (7)
N3	0.0203 (8)	0.0300 (9)	0.0182 (7)	−0.0007 (7)	0.0008 (6)	−0.0010 (6)
C1	0.0205 (9)	0.0234 (9)	0.0186 (8)	0.0022 (7)	0.0016 (7)	0.0004 (7)
C2	0.0138 (9)	0.0243 (9)	0.0191 (8)	0.0035 (7)	0.0011 (6)	0.0020 (7)
C3	0.0195 (10)	0.0239 (9)	0.0245 (9)	0.0002 (8)	0.0013 (7)	−0.0004 (7)
C4	0.0238 (10)	0.0243 (9)	0.0278 (9)	0.0030 (8)	0.0062 (8)	0.0069 (8)
C5	0.0252 (10)	0.0331 (11)	0.0178 (8)	0.0079 (8)	0.0043 (7)	0.0052 (8)
C6	0.0237 (10)	0.0276 (10)	0.0197 (8)	0.0048 (8)	−0.0005 (7)	−0.0048 (7)
C7	0.0162 (9)	0.0212 (9)	0.0226 (9)	0.0026 (7)	0.0028 (7)	0.0002 (7)
C8	0.0182 (9)	0.0197 (9)	0.0191 (8)	0.0001 (7)	−0.0012 (7)	0.0003 (7)
C9	0.0162 (9)	0.0172 (9)	0.0254 (9)	0.0003 (7)	0.0029 (7)	−0.0016 (7)
C10	0.0164 (9)	0.0249 (10)	0.0236 (9)	0.0019 (7)	−0.0038 (7)	0.0003 (7)
C11	0.0217 (9)	0.0230 (9)	0.0187 (8)	0.0008 (8)	−0.0030 (7)	0.0015 (7)
C12	0.0196 (9)	0.0186 (8)	0.0192 (8)	−0.0008 (7)	0.0018 (7)	−0.0015 (7)
C13	0.0153 (9)	0.0238 (9)	0.0191 (8)	−0.0009 (7)	−0.0012 (6)	−0.0010 (7)

### Geometric parameters (Å, °)

**Table:**

Cl1—C9	1.7411 (18)	C4—C5	1.386 (3)
O1—C1	1.216 (2)	C4—H4A	0.9500
O2—N2	1.231 (2)	C5—C6	1.383 (3)
O3—N2	1.223 (2)	C5—H5A	0.9500
O4—N3	1.223 (2)	C6—C7	1.384 (2)
O5—N3	1.2240 (19)	C6—H6A	0.9500
N1—C1	1.362 (2)	C8—C13	1.395 (2)
N1—C8	1.403 (2)	C8—C9	1.401 (2)
N1—H1A	0.8800	C9—C10	1.383 (2)
N2—C7	1.462 (2)	C10—C11	1.383 (3)
N3—C12	1.469 (2)	C10—H10A	0.9500
C1—C2	1.512 (2)	C11—C12	1.381 (3)
C2—C3	1.388 (3)	C11—H11A	0.9500
C2—C7	1.391 (3)	C12—C13	1.380 (2)
C3—C4	1.390 (2)	C13—H13A	0.9500
C3—H3A	0.9500		
			
C1—N1—C8	127.64 (15)	C5—C6—C7	118.53 (17)
C1—N1—H1A	116.2	C5—C6—H6A	120.7
C8—N1—H1A	116.2	C7—C6—H6A	120.7
O3—N2—O2	124.13 (17)	C6—C7—C2	122.60 (17)
O3—N2—C7	118.45 (16)	C6—C7—N2	117.81 (16)
O2—N2—C7	117.41 (16)	C2—C7—N2	119.51 (16)
O4—N3—O5	123.50 (16)	C13—C8—C9	118.04 (16)
O4—N3—C12	118.03 (14)	C13—C8—N1	123.03 (16)
O5—N3—C12	118.46 (15)	C9—C8—N1	118.92 (16)
O1—C1—N1	124.98 (16)	C10—C9—C8	122.07 (16)
O1—C1—C2	121.51 (16)	C10—C9—Cl1	118.51 (14)
N1—C1—C2	113.45 (15)	C8—C9—Cl1	119.41 (13)
C3—C2—C7	117.83 (16)	C11—C10—C9	119.91 (17)
C3—C2—C1	120.22 (16)	C11—C10—H10A	120.0
C7—C2—C1	121.73 (16)	C9—C10—H10A	120.0
C2—C3—C4	120.48 (17)	C12—C11—C10	117.65 (16)
C2—C3—H3A	119.8	C12—C11—H11A	121.2
C4—C3—H3A	119.8	C10—C11—H11A	121.2
C5—C4—C3	120.34 (17)	C13—C12—C11	123.77 (17)
C5—C4—H4A	119.8	C13—C12—N3	117.93 (16)
C3—C4—H4A	119.8	C11—C12—N3	118.28 (15)
C6—C5—C4	120.21 (16)	C12—C13—C8	118.55 (17)
C6—C5—H5A	119.9	C12—C13—H13A	120.7
C4—C5—H5A	119.9	C8—C13—H13A	120.7
			
C8—N1—C1—O1	6.7 (3)	O2—N2—C7—C2	−29.6 (2)
C8—N1—C1—C2	−176.12 (17)	C1—N1—C8—C13	−7.6 (3)
O1—C1—C2—C3	110.1 (2)	C1—N1—C8—C9	171.65 (18)
N1—C1—C2—C3	−67.2 (2)	C13—C8—C9—C10	1.0 (3)
O1—C1—C2—C7	−64.4 (3)	N1—C8—C9—C10	−178.25 (16)
N1—C1—C2—C7	118.28 (19)	C13—C8—C9—Cl1	−179.73 (13)
C7—C2—C3—C4	−0.8 (3)	N1—C8—C9—Cl1	1.0 (2)
C1—C2—C3—C4	−175.50 (17)	C8—C9—C10—C11	−0.8 (3)
C2—C3—C4—C5	1.0 (3)	Cl1—C9—C10—C11	179.93 (14)
C3—C4—C5—C6	−0.3 (3)	C9—C10—C11—C12	0.5 (3)
C4—C5—C6—C7	−0.5 (3)	C10—C11—C12—C13	−0.4 (3)
C5—C6—C7—C2	0.8 (3)	C10—C11—C12—N3	178.34 (16)
C5—C6—C7—N2	−175.84 (17)	O4—N3—C12—C13	−2.9 (2)
C3—C2—C7—C6	−0.1 (3)	O5—N3—C12—C13	177.27 (17)
C1—C2—C7—C6	174.54 (17)	O4—N3—C12—C11	178.28 (18)
C3—C2—C7—N2	176.43 (16)	O5—N3—C12—C11	−1.6 (3)
C1—C2—C7—N2	−8.9 (3)	C11—C12—C13—C8	0.7 (3)
O3—N2—C7—C6	−31.8 (2)	N3—C12—C13—C8	−178.11 (15)
O2—N2—C7—C6	147.07 (18)	C9—C8—C13—C12	−0.9 (2)
O3—N2—C7—C2	151.52 (17)	N1—C8—C13—C12	178.32 (16)

### Hydrogen-bond geometry (Å, °)

**Table:**

*D*—H···*A*	*D*—H	H···*A*	*D*···*A*	*D*—H···*A*
C13—H13A···O1	0.95	2.24	2.848 (2)	121
C10—H10A···O4^i^	0.95	2.35	3.246 (2)	157
C11—H11A···O2^ii^	0.95	2.55	3.202 (2)	126

Symmetry codes: (i) *x*+1, *y*, *z*; (ii) *x*+1/2, −*y*+1/2, −*z*+1.
