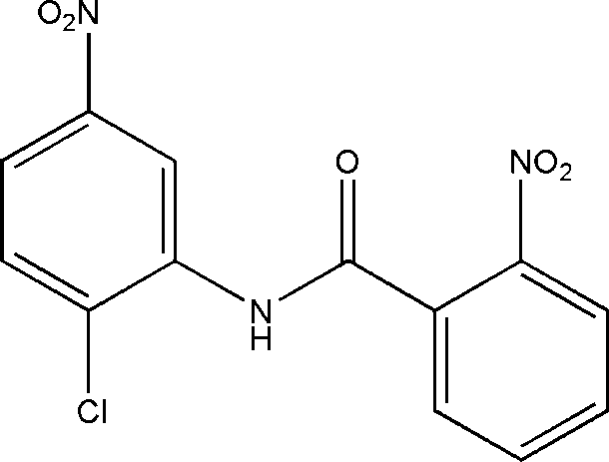# *N*-(2-Chloro-4-nitro­phen­yl)-2-nitro­benzamide. Corrigendum

**DOI:** 10.1107/S1600536808013949

**Published:** 2008-06-07

**Authors:** Aamer Saeed, Shahid Hussain, Ulrich Flörke

**Affiliations:** a Department of Chemistry, Quaid-i-Azam University, Islamabad, Pakistan; b Department Chemie, Fakultät für Naturwissenschaften, Universität Paderborn, Warburgerstrasse 100, D-33098 Paderborn, Germany

## Abstract

The title and the chemical diagram of the paper by Saeed, Hussain & Flörke [*Acta Cryst.* (2008), E**64**, o705] are corrected.

## Abstract 2

Corrigendum to *Acta Cryst.* (2008), E**64**, o705.

In the paper by Saeed, Hussain & Flörke [*Acta Cryst.* (2008), E**64**, o705], the title and the chemical diagram are incorrect. The correct structure is shown below and the correct title of the original paper should be ‘*N*-(2-Chloro-5-nitro­phen­yl)-2-nitro­benzamide’.